# Hyperthermal-driven mass extinctions: killing models during the Permian–Triassic mass extinction

**DOI:** 10.1098/rsta.2017.0076

**Published:** 2018-09-03

**Authors:** Michael J. Benton

**Affiliations:** School of Earth Sciences, University of Bristol, Bristol BS8 1RJ, UK

**Keywords:** hyperthermal, Permian–Triassic mass extinction, hypoxia, hypercapnia, global warming, acid rain

## Abstract

Many mass extinctions of life in the sea and on land have been attributed to geologically rapid heating, and in the case of the Permian–Triassic and others, driven by large igneous province volcanism. The Siberian Traps eruptions raised ambient temperatures to 35–40°C. A key question is how massive eruptions during these events, and others, could have killed life in the sea and on land; proposed killers are reviewed here. In the oceans, benthos and plankton were killed by anoxia–euxinia and lethal heating, respectively, and the habitable depth zone was massively reduced. On land, the combination of extreme heating and drought reduced the habitable land area, and acid rain stripped forests and soils. Physiological experiments show that some animals can adapt to temperature rises of a few degrees, and that some can survive short episodes of increases of 10°C. However, most plants and animals suffer major physiological damage at temperatures of 35–40°C. Studies of the effects of extreme physical conditions on modern organisms, as well as assumptions about rates of environmental change, give direct evidence of likely killing effects deriving from hyperthermals of the past.

This article is part of a discussion meeting issue ‘Hyperthermals: rapid and extreme global warming in our geological past’.

## 1. Introduction

Mass extinctions have been much discussed since 1980, and most attention has focused on the ultimate drivers of the physical environmental crisis rather than the proximate killers of life. The earlier tension between impact and volcanism models has simplified, with a realization that asteroid impact might have been a prime driver only of the mass extinction at the end of the Cretaceous, 66 Myr ago. Further, it is becoming clear that hyperthermals, environmental breakdowns driven by massive volcanic eruptions, were likely the cause not only of the mass extinctions at the ends of the Permian and Triassic periods, but of many more events. The aim here is to explore how hyperthermals kill plants and animals, both in the sea and on land, using the Permian–Triassic mass extinction as the exemplar event, because it has been most studied, but it provides models applicable to all hyperthermal-driven crises on Earth.

The idea that large igneous province (LIP) volcanism might have caused mass extinctions emerged through the 1980s and 1990s, when Rampino & Stothers [1] and Courtillot [2] documented the coincidence of timing between phases of LIP volcanism and mass extinctions. The link between volcanism and killing had in fact been noted earlier, when Vogt [3] suggested that the eruption of the Deccan Traps in India had triggered the Cretaceous–Palaeogene mass extinction (KPgME) by poisoning life with trace metals. Other killing agencies explored in those early days included global cooling, even mini ice ages, set off by the release of sulfate aerosols [4]. The debate became mainstream following the publication of the classic Alvarez paper in 1980 [5], which provided evidence for impact as the driver of the KPgME, and triggered a continuing and heated debate about the relative merits of massive volcanism versus asteroid impact as the main killing agents in this crisis, and others.

Whereas some argued that asteroid impact was the main driver of mass extinctions, and that such impacts even followed a regular periodicity [6], that view has been largely abandoned through lack of evidence [7]. In a recent review of mass extinctions, Bond & Grasby [8] identify five major global extinction events and 12 other mass extinctions through the past 500 Myr. Of these 17 killing events, four are associated with coeval large craters, but only the Chicxulub is a convincing driver for the KPgME, and there are serious doubts about the validity of the others. These authors [8] note also that there is independent evidence of global cooling associated with three of the older events, both cooling and warming associated with four more events, neither with one, and global warming with nine events, including the Capitanian, Permian–Triassic (PTME), Smithian–Spathian, Carnian, end-Triassic (ETME) and early Toarcian extinction events, a series spanning part of the Permian and the early Mesozoic, and dated, respectively, at 260, 252, 249, 232, 201 and 183 Ma. This Permian to early Mesozoic interval was a time of globally warm temperatures, no icecaps and the single supercontinent Pangaea [9]. It was also a time of major upheaval in life, with the demise of the so-called Palaeozoic faunas in the oceans and on land, and the rise of modern ecosystems [10,11].

These six extinction events through the Permian to Early Jurassic interval are all now most plausibly explained as driven by the consequences of LIP volcanism, being associated in turn [8,9] with the Emeishan Traps in China (Capitanian), the Siberian Traps in Russia (PTME, Smithian–Spathian), the Wrangellia basalts in western North America (Carnian), the Central Atlantic Magmatic Province (ETME) and the Karoo–Ferrar basalts of South Africa (early Toarcian). Each was associated with substantial global extinction on land and in the sea, and the driving model in each case was associated with global warming, acid rain and ocean acidification. The largest of these six events was the PTME, and this has been the subject of most research and the source of detail about the favoured killing model.

## 2. The Permian–Triassic mass extinction

The PTME comprised two killing events, one at the very end of the Permian (EPME) and a second at the beginning of the Triassic, separated by 60 000 years [12]. Together, these pulses of extinction accounted for the loss of up to 96% of marine invertebrate species globally [6], and similar losses at regional scale, when documented in detail in marine sections in South China [13,14]. Species losses of plants and animals on land were probably comparable, although the data are harder to compile [15,16], but the consequences for life both on land and in the sea were profound changes in ecosystems and in the ecologically dominant groups.

The PTME was quantitatively, but also qualitatively, more severe than any other Phanerozoic mass extinction, and its effect in resetting evolution was noted early in the days of statistical palaeontology [17], and this has never been doubted. In the sea, Palaeozoic ecosystems based on rugose and tabulate corals, brachiopods, crinoids and ganoid-scaled fishes (as well as graptolites and trilobites, which had died out earlier) were replaced by ‘Modern’ ecosystems dominated by scleractinian corals, molluscs, malacostracan arthropods (crabs, lobsters), echinoids and neopterygian fishes [8–11]. These new groups had been identified as part of the Mesozoic marine revolution (MMR), a time of changing faunas and accelerating predator–prey interactions, a kind of macroecological arms race [18]. Extensive new evidence from China and elsewhere suggests that the MMR might well have begun during the turbulent phase of the recovery of life following the PTME [19,20], with evidence of exceptional faunas comprising modern faunal components such as malacostracans, neopterygian fishes and marine reptiles already abundant and diverse by the Anisian (Middle Triassic, 8 Myr after the PTME).

On land too, the PTME and Early to Middle Triassic recovery episode marked a major punctuation in the history of life. Changes in plants and insects are hard to document [16]. Among plants, for example, we know that ecological impacts were profound, with major changes in floras, the proliferation of disaster species such as *Pleuromeia* in the aftermath of the event and a 10 Myr ‘coal gap’, a time when there were no trees or forests. Stable gymnosperm-dominated Permian floras were replaced by rapidly growing, early successional communities dominated by lycopods and ferns in the earliest Triassic of the Northern Hemisphere. There was indeed a major loss of plant diversity, with extinctions of families of non-seed plants and conifers [21]. Insect extinctions are harder to document as data are incomplete, but there was a major turnover in entomofaunas through this interval, during which the Carboniferous and Permian fauna of herbivorous arthropods (mites and apterygote and basal pterygote insects) was replaced by the modern phase (Triassic to Recent) comprising mites, orthopteroids, hemipteroids and basal holometabolans [22]. Among tetrapods, late Palaeozoic faunas of lumbering pareiasaurs and therapsids, such as dinocephalians, anomodonts and gorgonopsians, gave way to modern terrestrial ecosystems comprising dinosaurs and pterosaurs, but also turtles, lizards, crocodiles and mammals, most of which eventually emerged in the Late Triassic [11,16].

**Figure 1.**
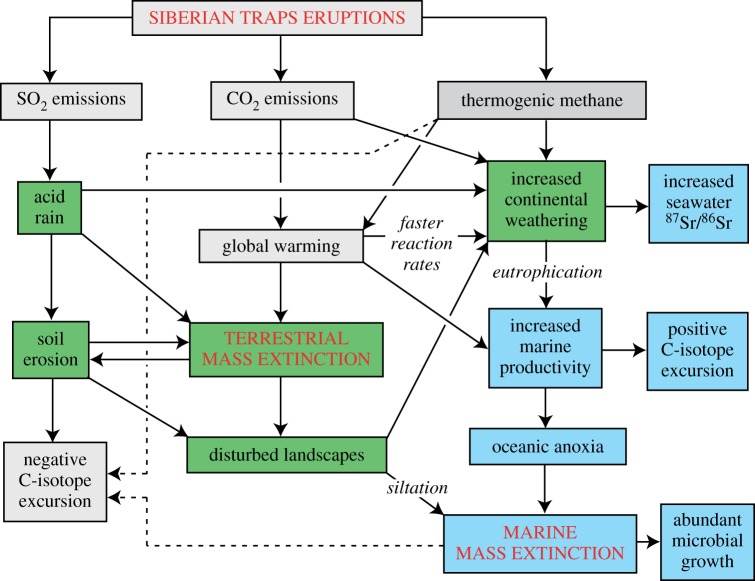
Model of likely environmental consequences of the Siberian Traps eruptions, showing the flows of consequences of global warming and acid rain. Causal links are indicated by solid arrows, and possible second-order controls on the negative carbonate C-isotope excursion at the EPME are indicated by dashed lines. Based on [23]. (Online version in colour.)

Although geologists and palaeontologists have identified multiple possible causes of the PTME, and evidence for asteroid impact is debatable, the majority view supports a cohesive model based on volcanic eruption, and particularly consequences deriving from the eruption of the massive Siberian Traps LIP, which dates precisely to this time [4,8,9,12]. The model (figure 1) includes acid rain, global warming and ocean anoxia. Sedimentary evidence highlighted the sudden switch from oxic to anoxic conditions in marine sediments worldwide. Stable isotope studies confirm this, and show repeated peaks of global warming, exactly at the Permo-Triassic boundary (PTB), and repeatedly through the Early Triassic. Decreases in the *δ*^18^O ratio of shallow marine biogenic carbonate and palaeosols suggest that seawater and soil temperatures may have risen by 8–10°C at the PTB, and a further 6–8°C during the Smithian, 1 Myr later. Temperatures at the Equator are estimated at 32–35°C in the earliest Triassic, and up to 40°C at the Smithian–Spathian boundary [24]. These elevated temperatures repeatedly drove life from tropical oceans and lands in complex ways, and must have directly caused some of the extinctions [24,25]. Further, first-hand geological evidence is that thick units of coarse-grained sediment appear exactly at the PTB in many terrestrial sedimentary basins, suggesting stripping of trees and soil and rapid erosion [26], and there is a silica spike in shallow marine sediments worldwide, indicating wash-off of sediment and plant debris exactly at the PTB [27].

Putting all these primary observations together, the standard model for LIP-induced mass extinction (figure 1) comprises a series of cause-and-effect processes. The SO_2_ emissions primarily, on mixing with water vapour in the atmosphere, caused acid rain, which in turn killed land plants and caused soil erosion. The CO_2_ emissions caused global warming, and the effect was enhanced by the consequent release of methane hydrate stores from frozen, deep-ocean settings. Together, the acid rain and global warming caused terrestrial extinctions. In addition, the increased continental weathering induced by acid rain and global warming led to increased marine productivity and eutrophication, and so oceanic anoxia, and marine mass extinctions.

This has become a ‘standard model’ for many other mass extinctions, most notably the ETME, and some others through the Permian, Triassic and Jurassic [8,9]. A key question then is how such a model, and especially the global warming aspect, kills life. We review suggested physical consequences of the hyperthermal model and evidence about their killing potential, in sea and on land.

## 3. Hyperthermals as killers in the sea

### (a) Killers in the sea

Numerous hyperthermal episodes in the history of the Earth have been associated with extinction, most notably the Palaeocene–Eocene Thermal Maximum (PETM; although extinction levels were minor, limited to pelagic benthic foraminifera), several ocean anoxic events through the Mesozoic, and the PTME [4,8,9,28]. The scaling and selectivity of such events have been explored, but it is difficult in all cases to identify the exact loss of biodiversity. Palaeontologists can estimate rates of species loss at either local or regional scale, where they document every fossil specimen through a measured rock section, and attempt to track the fate of each species. Such studies are not easy, as absence of a fossil lineage could mean extinction or could simply mean a failure in preservation or collection. On all scales, but especially the global scale, preservation and collection failures may bias the measurements of extinction magnitudes. Multiple sections worldwide through the same event can provide some corroboration of general trends. These studies show that for a truly major event such as the PTME, the rate of species extinction can be greater than 90% [13], and for smaller events such as the PETM, the rate of loss regionally can be much smaller, with turnovers of 1–20% of species.

Selectivity of taxa during extinction events can indicate causality. If, for example, most of the victims share certain physiological properties, then that might identify the killing stresses. The most convincing such efforts have been done around the PTME, for which meta-analytical studies of marine animals [29,30] suggested that physiologically poorly buffered species suffered statistically significantly more through the event than well-buffered species. Poorly buffered species include corals, sponges, brachiopods, bryozoans and crinoids, whereas well-buffered groups include bivalves, gastropods, cephalopods, ostracods and trilobites. The classification of survivors and victims is not perfect, however, as gastropods and ostracods, while ‘poorly buffered’ actually were great survivors during the PTME, and the ‘well-buffered’ ammonoids suffered severe extinctions [31].

The argument [29,30] was that well-buffered animals are adapted to withstand the stresses of increased *p*CO_2_ and temperature and reduced *p*O_2_ and carbonate saturation, all associated with marine anoxia and ocean acidification. Marine animals with weakly buffered respiratory physiology are less able to control their intracellular pH and so maintain respiratory efficiency under elevated CO_2_ levels than well-buffered organisms, which means they are more susceptible to extinction from hypercapnia or ocean acidification [29,30]. Skeleton chemistry was important also: species with phosphatic or siliceous skeletons (linguliform brachiopods, conulariids, and hexactinellid and non-hypercalcified demosponges) were significantly more likely to survive the PTME than those with calcareous skeletons, whether aragonite or calcite, even when accounting for differences in physiology, geographical range and other traits [30]. An alternative model [31] includes the role of acidification, but also the idea that the habitable zone in the oceans was substantially reduced by warming from above and anoxia from below, and that there was no single key driver such as hypercapnia.

### (b) Hypoxia as a killer in the sea

In a physiological context, hypoxia is ‘deficiency in the amount of oxygen reaching the tissues’, and this is a well-known stressor of modern organisms. Oxygen levels in the atmosphere and surface ocean waters have varied substantially through the Phanerozoic, ranging from 13% to 31%, compared to a modern value of 21% [32]. Atmospheric oxygen levels fell from a high point of 28% in the Late Permian to 20% at the PTB, and 16% in the Early Triassic [32].

In experimental situations, many marine organisms exhibit tolerance to hypoxia when compared with terrestrial organisms [33]. Oxygen content of seawaters varies enormously in modern oceans, with oxygen minimum zones (OMZs) in warmer surface waters, sometimes linked with eutrophication, and at depth in upwelling zones, sometimes with less than 10% of surface oxygen content in permanent oxygen-deficient layers. When oxygen conditions are insufficient to support life, animals move up and down in the water column to layers with sufficient oxygen, or laterally and even latitudinally to water bodies at the correct depth but with sufficient oxygen [34].

At times of climate change, hypoxia-intolerant species move away from waters that develop low oxygen levels, and their overall distributions can be compressed, so leading to competition for food or shelter, or a net loss in species diversity and ecosystem function [34]. In modern examples, hypoxia-tolerant species can survive at the expense of those that require higher oxygen levels. Larval life-history stages tend to be intolerant to hypoxia, and hypoxia can also select for smaller adult individuals and lower overall biomass. At ecosystem scale, there may be a marked shift in energetics with continued oxygen loss as the overall metabolism of the system reduces; alternative electron receptors replace oxygen in the system and these yield less energy at the base of the food web [34]. In studies of modern fisheries, sustained reduction in oxygen over many decades reduces the overall biomass of fishes retrieved from the seabed as a result of switching carbon transfer to secondary production. On geological time scales, it is likely that the same phenomenon would readily occur.

Marine biologists and fisheries experts define several hypoxia thresholds that mark important shifts in ecosystem function [35]. Compared to normal levels of oceanic oxygenation (greater than 1.4 ml l^−1^), hypoxia is defined as anything less than this (less than 1.4 ml l^−1^), and severe hypoxia at much lower levels (less than 0.5 ml l^−1^). These concepts, and the whole-ecosystem approach in fisheries research, are useful for ecologists and palaeontologists because all species show different responses to hypoxia, and some even show different responses at different developmental stages, and further, these responses depend on other conditions, such as salinity, productivity and especially water temperature [36]. Therefore, it would be hard to predict a general response of any species or clade to hypoxia at different intensities. In addition, most physiological experiments have been under controlled laboratory conditions, where responses might not reflect the complexities of Nature [32,34].

In the end, it seems unlikely that hypoxia could have driven the PTME because the rate of change in oxygen levels, reflecting the huge volume of oxygen in the atmosphere, is inevitably slow, lasting millions or tens of millions of years, and so allowing organisms plenty of time to adapt. Further, the fluctuations during the Late Permian and Early Triassic were not outside the scope of modern oxygen levels at different altitudes, to which life has adapted. Therefore, although hypoxia has been cited as a key killer during the PTME crisis [29–32], further evidence for rapid fluctuations would be required to make a case.

### (c) Hypercapnia and ocean acidification as killers in the sea

Hypercapnia is the physiological condition of retaining too much carbon dioxide in the blood, and it can be induced by medical problems or by an inappropriate external environment. It has been argued that the main killer in the PTME oceans was hypercapnia associated with acidification, hypoxia and toxic sulfide levels [29], but this has been queried [31]. Excess CO_2_ in seawater can have severe physiological effects on organisms, suppressing their metabolism, disrupting acid–base homeostasis and impairing calcification through reduced mineral saturation [36,37]. However, predicted outcomes of these effects are not as clear as might have been expected through the PTME [31].

It has been suggested [29,30] that much of the relative survivorship of major marine animal groups through the PTME can be explained by the impact of hypercapnia on their ability or need to construct a carbonate skeleton. Worst affected (86% generic extinction rate) were the poorly buffered forms with a high need for carbonate ions, such as rugose corals, rhynchonelliform brachiopods and crinoids. Next worst affected (54% generic extinction rate) were the well-buffered forms that needed lower amounts of carbonate for their shells, such as gastropods, bivalves, nautiloids, ammonoids, ostracods and echinoids. Finally, the physiologically well-buffered forms that required little or no carbonate (5% generic extinction rate) were the ctenostome bryozoans, lingulid brachiopods, holothurians, conodonts and chondrichthyan fishes.

In a test based on fish evolution through the Permian and Triassic [38], fewer chondrichthyan fishes suffered extinction than marine invertebrates, which was interpreted as evidence that sharks and their allies are active aerobic organisms with high aerobic scope for thermal tolerance and well-developed acid–base regulation, traits that gave them resilience to oceanic hypercapnia at the PTME. By contrast, bony fishes (osteichthyans) seemingly underwent extinctions comparable to those of marine invertebrates, perhaps reflecting their differing physiologies. The validity of such studies on fish evolution through the PTME is uncertain, however, as the record is poor around the PTB, and others see little evidence of extinction among fish groups.

The role of hypercapnia as a PTME killer has been seriously questioned [31]. The argument is that hypercapnia resistance should be seen best in burrowing taxa, which would regularly encounter high levels of CO_2_ in the sediment, but it is not. Comparisons of extinction likelihoods had shown [30] that infaunal bivalves were especially likely to go extinct, whereas they should be best adapted to survive hypercapnia. These authors [31] note also that the survival of poorly buffered gastropods and ostracods, and extinction of well-buffered ammonoids, as well as the sponges and radiolarians with silicified skeletons, also speaks against the importance of hypercapnia as a killer.

### (d) Global warming as a killer in the sea

The third component of environmental change that would have acted as a killer during the PTME is increased global temperature. As noted, oxygen isotopes show rises of 8–10°C at the PTB, and during repeated warming episodes through the Early Triassic [4,8,9,24]. Today, organisms all have preferred temperature ranges. Further, many features of biology and ecology relate to temperature, such as body size and total diversity and productivity of ecosystems. As with hypoxia and hypercapnia, organisms move away from unfavourable conditions. In recent times, it is well documented how species of animals and plants migrate to maintain themselves in favourable conditions even with temperature changes of as little as ±1°C. The nature and timing of temperature change can be as important as the magnitude of the change. For example, some corals can increase their thermal tolerance by 1–1.5°C, and this adaptation might reduce their vulnerability to bleaching events by some 30–50 years [39].

The rate of warming is also crucial. Experiments with various species show that a reduction in the rate of temperature rise can reduce the upper temperature tolerance limit [40]. This might seem counterintuitive, that the rate of warming is proportional to temperature tolerance: higher temperature tolerances are achieved in experiments when the rate of warming is fast. This is because there are two factors in play, the critical maximum temperature at which cellular and biochemical processes fail, and the maximum temperature to which a species can become acclimatized [40]. During a rapid pulse of heating, an organism might survive for a short time, whereas longer-term warming involves acclimatization and adaptation of physiological processes. During fast warming, species can survive at higher temperatures before anaerobic end-product accumulation overcomes resistance capacity, a process termed hardening. At slow rates of warming, over months or years, and where the upper limits are well below the critical oxygen limits, acclimatization and adaptation effects become important. Long-term thermal limits are significantly lower than medium-term survival values [40]. This suggests that many organisms might survive a very short, but acute, episode of global warming, lasting for days or weeks at most, but would succumb if the elevated temperature was maintained for months or years, as in ancient examples such as the PTME.

### (e) Interactions of multiple stressors

It is well known in physiological studies that stressors can interact in complex ways [36,41]. For example, in experimental studies on marine organisms, elevated CO_2_ can reduce the upper thermal limits for a species, and exacerbate the effects of low pH on lowering metabolic rate [41]. Likewise, ocean acidification can affect the ability of organisms to survive elevated temperatures. In other cases, changing temperature or pH can have measurable deleterious effects, which can then be multiplied when they act together [41]. These synergistic effects mean that the lethal temperature for most animals is 35°C, because oxygen demand increases with temperature [37]. This causes hypoxaemia and the onset of anaerobic mitochondrial metabolism that is only sustainable for short periods.

**Figure 2.**
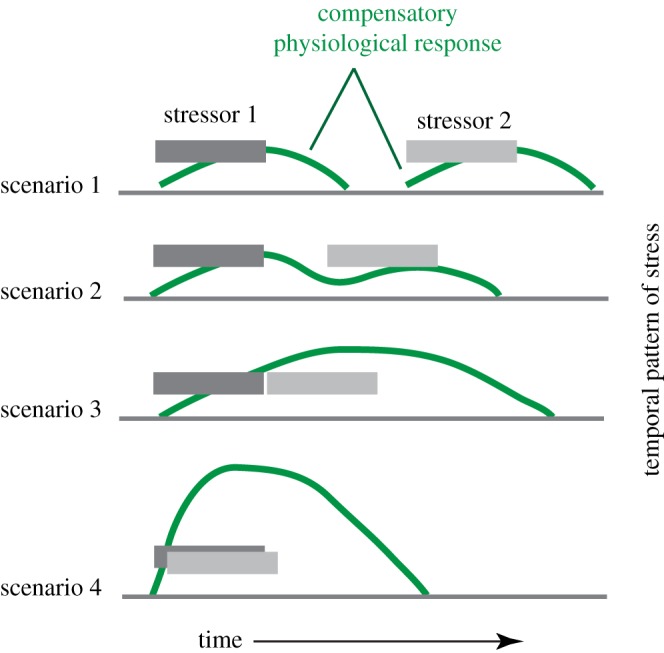
A model to represent how multiple stressors can interact, showing two hypothetical stressors, as dark grey and light grey bars. Four scenarios show stressors occurring closer and closer together in time, and with responses ranging from no interaction (scenario 1) to antagonistic, where the first stressor reduces the impact of the second stressor (scenario 2), to synergistic, where the response to both stressors can produce an additive response (scenarios 3 and 4). The areas under the physiological response curves represent the total physiological impact of stress in terms of the energetic expenditure required to return to homeostasis (dark grey horizontal lines). Based on data in [41]. (Online version in colour.)

Reactions to different stressors may be similar in terms of cellular protection, and so adaptation to one stressor, such as acute heat stress, can mean the organism responds better to a subsequent osmotic, chemical or acidification stress [41]. Such interactions of stressors can either improve or reduce tolerance for other stressors [41]. In four possible scenarios (figure 2), an initial stress, such as a sharp increase in temperature or reduction in oxygen, can occur so long before a second stress that the organism recovers to normal, and so responds as if unprepared to a second stress (scenario 1). In the other scenarios, the stresses occur close enough together temporally that the organism is primed to resist stress, and the effect of the second stressor is reduced (scenario 2). If the second stressor follows too rapidly, however, the organism may not yet have adapted to the first stressor, and so the two stressors interact as if they are a single external forcing agent (scenario 3) or they even interact synergistically to produce an overall increased response (scenario 4).

Adaptation to stress and the development of cross-tolerance, where one stress enhances the response to a second, later stress, are often mediated by heat shock proteins (HSPs). The time scale of HSP expression is rapid, with significant HSP upregulation occurring within 0.5–6 h, and with peak expression typically within 15 h of the initial exposure. Usually, HSP expression dwindles after that, if the stressor has been removed, and returns to control levels by 24–48 h after the stimulus [41]. These timings suggest the likely nature of responses to multiple stressors in the models (figure 2). If sharp changes in temperature, hypercapnia or hypoxia occur over short time scales (from hours to days), then cross-tolerance may develop; this applies, for example, to cases of daily heat and hypoxia stress in tidal pools. If the changes are on longer time scales, such as monthly or annual changes in seasonal conditions, then cross-tolerance is unlikely, and effects of stressors will be additive. In the case of a hyperthermal event, where heat stress, hypoxia and hypercapnia might all occur together, synergies such as interaction and cross-tolerance would have occurred.

### (f) The ‘double whammy’

Song *et al*. [31], in arguing against the idea that marine survivors were well buffered against hypercapnia and ocean acidification [29,30], presented their ‘double whammy’ model in which marine life was not only driven from the tropical seas by extreme warming, but then the liveable zone was massively restricted by lethal warming from above and anoxia/euxinia from below. The ‘double whammy’ consists of these two drivers, which resulted in a habitable zone of nothing at all in shallow tropical waters, and a habitable refuge zone of as little as 10% of the normal depth range elsewhere. Further, benthic organisms would have been killed worldwide by anoxic–euxinic seabed conditions, and plankton and surface nekton by heating from above.

## 4. Hyperthermals as killers on land

### (a) Killers on land

Hypoxia and global warming have been cited as killers of life on land at the PTME. However, the hyperthermal event triggered many other physical environmental catastrophes that might have played as important a role, including aridity, acid rain and mass wasting, wildfires and opening of an ozone hole [16]. Establishing which of these might have been important is hard because there have been no meta-analytical studies of terrestrial victims and survivors through the PTME analogous to the comparisons of well- and poorly buffered marine taxa [29–31]. The arguments then revert to the circumstantial, namely considering what happens in modern plants and animals, issues concerning rates of change and whether any anecdotal fossil evidence can be identified to support a particular case.

### (b) Hypoxia as a killer on land

Of the marine killers, hypoxia has been cited most often as having been important also on land [32,42,43]. The focus has been on insects and vertebrates, whose existence was supposedly curtailed by the reduced oxygen levels coincident with the PTME. For plants, the effects of low oxygen are complex; experiments show that hypoxia generally reduces vegetative growth in C_3_ plants, but not in C_4_ plants, and it can decrease seed growth.

Physiological experiments on modern insects show that they can escape ill effects of hypoxia by moving to areas of higher oxygen, or by opening spiracles and pumping, so more oxygen reaches their tissues [44]. Further, many insects can tolerate a wide range of oxygen partial pressures in their muscles. Hypoxia causes physical adaptation, such as increasing tracheal diameter or the penetration of trachea into tissues, or increasing haemoglobin levels in the blood, all of which enable them to extract more oxygen [44]. Insects can also switch to anaerobic pathways for ATP production, and reduce oxygen demand by reducing body size by reducing development time, growth rate and fecundity. Modern insects encounter hypoxia in aquatic habitats (evaporating pools), in soils and flooded burrows, and at high altitudes.

Similarly, vertebrates can adapt to hypoxia by reducing growth rates and body size, and by improving lung and gill structures and haemoglobin volumes to improve oxygen extraction efficiency. Ectothermic fishes, amphibians and reptiles are generally better able to adapt to hypoxia than endotherms such as mammals and birds, because of their very different metabolic demands [45]. The degrees of tolerance to hypoxia can vary enormously, with some reports of almost miraculous feats of survival by carp, crocodiles and turtles in frozen ponds. More usually, modern ectothermic vertebrates can tolerate hypoxia by making long-term physiological adaptations by suppressing their metabolism, being able to tolerate metabolite accumulation, and in establishing free-radical defences during reoxygenation [45]. In all these cases, however, the insects or vertebrates are responding to relatively short-term experimental stimuli, and so the effects of a longer-term fall in oxygen levels are harder to predict. If the level was low, but not unsustainable, then some taxa would be driven to extinction by the stress, and others might adapt by modifying their respiratory anatomy and biochemistry, and by reducing body size.

In the case of the PTME, some palaeontologists [42,43] have pointed to real physical changes in the reptiles of the day. For example, they noted that the anomodont *Lystrosaurus*, which was a famous survivor, had a barrel-like chest to accommodate expanded lungs, perhaps a muscular diaphragm to force air in and out of the lungs more speedily, and a possible four-chambered heart to improve the efficiency of blood circulation. In addition, late Permian and Triassic therapsids had nasal turbinates, thin bone lamellae within the nasal cavity that substantially increase the area of nasal mucous membranes, possibly to enhance oxygen uptake, to assist with countercurrent heat exchange or both. It has also been noted that, in anomodonts, the secondary palate and the internal nostrils were enlarged, both supposedly to improve oxygen uptake. These are all ingenious observations, but they could all have other explanations, such as a switch in physiology from ectothermic to endothermic, or changing body size. Further, the rate of change in oxygen levels in the atmosphere was likely exceptionally slow [32], and quite inadequate to cause serious levels of extinction.

### (c) Global warming as a killer on land

There is evidence for substantial temperature change through the PTME and Early Triassic crises. The first effect on life on land was surely migration, as plants and animals fled the tropics to more congenial locations [24,25]. In a study based on both skeletal and footprint data, details of these migrations were detected [25], with a clear geographical disjunction through the PTME, with tetrapod distribution shifting 10–15° poleward. There was then a rapid expansion phase across the whole of Pangaea following the PTME. These changes support a model of generalized migration of tetrapods to higher latitudinal, cooler regions, to escape from the superhot equatorial climate in the earliest Triassic [24], but the effect was shorter in duration, and not as pronounced as had been proposed.

For those plants and animals that did not move, high temperatures of 35–40°C would be lethal for most. In C_3_ plants, photorespiration replaces photosynthesis at temperatures over 35°C and few plants can survive above 40°C. In trees, heat stress reduces photosynthesis, increases photo-oxidative stress, causes leaves to burn and fall and reduces overall growth rates [46]. In some species, stomatal conductance increases at high temperatures, which may be a mechanism for leaf cooling. Heat tolerance in trees varies enormously depending on species, and even between individuals of a species. Elevated atmospheric CO_2_ can mitigate heat stress in certain cases. For most animals, temperatures of 40°C or higher can cause protein damage that can be countered only for short heat shocks by the production of heat-shock proteins [47].

The combined effects of extreme high temperatures and drought were studied in the European drought of summer 2003. For three months, leaf temperatures commonly exceeded 40°C, and, during these times, photosynthesis was reduced, mainly by biochemical limitations rather than closure of stomata [46]. Concentrations of carotenoids increased in leaves to improve protection against photo-oxidative damage. Other changes included reduction in cholorophyll concentration, closure of stomata and early senescence of leaves, presumably to protect the tree's hydraulic system by reducing transpiration. Many species of trees readily survived several months of extreme high temperatures and drought by ceasing growth [46], but it is not clear how long they could suspend their normal systems under prolonged heat stress.

For many animals, there is a critical temperature limit beyond which their physiological systems are compromised. From 35 to 40°C, most animals become uncomfortable, and seek to remove themselves from those high temperatures. For most metazoans, the absolute upper lethal temperature limit is said to be 47°C [36], and responses of animals on land can be observed today in desert studies. In Saudi Arabia, for example, the ambient temperature often reaches 45°C, and in summer, soil surface temperatures can even exceed 60°C. At temperatures above 35–40°C, respiratory evaporative water loss increases markedly, and yet this physiological cooling mechanism is full of danger as high environmental temperatures are usually associated with low water availability.

### (d) Aridity as a killer on land

Today, extreme high temperatures on land are often associated with drought, which generally exacerbates the challenge for trees [46]. A normal response to high temperature would be to open the stomata and increase transpiration and physical cooling of leaves; drought prevents this response.

**Figure 3.**
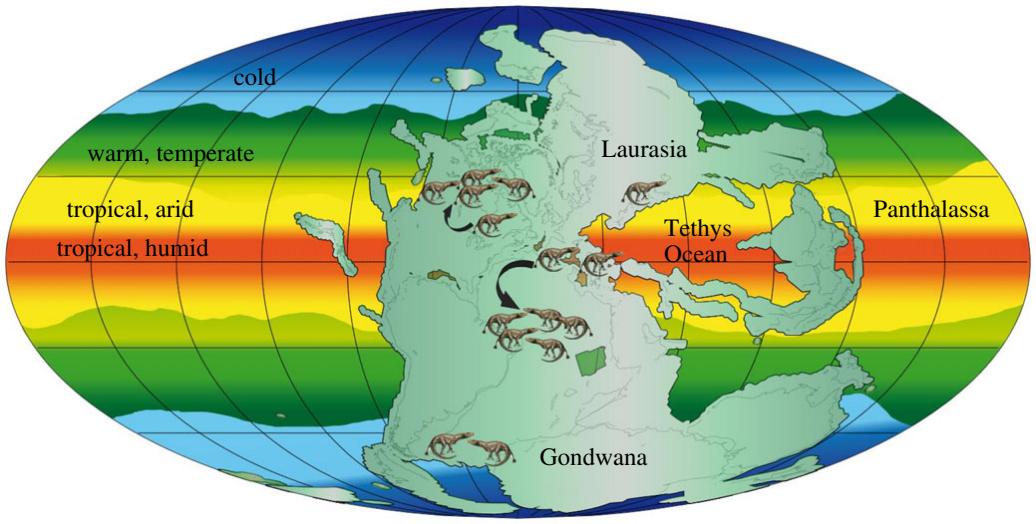
Palaeogeographic map of the Permo-Triassic, showing the single supercontinent Pangaea, modelled climate belts, and the distributions of terrestrial tetrapods. Tetrapods were distributed worldwide before and after the PTME crisis, but moved 10–15° polewards as a result of tropical overheating. The shift north is documented, but the fossil record is not good enough to document the Southern Hemisphere action. Key climatic belts, oceans and land masses are indicated. Image by Massimo Bernardi, MUSE, Trento, Italy. (Online version in colour.)

Permian–Triassic climates were generally warm. The supercontinent Pangaea extended from pole to pole, and a deep oceanic gulf, Tethys, split the supercontinent around the Equator (figure 3). Climate modelling [48,49] of this unusual continental and oceanic configuration highlights the broad tropical belt with a strong monsoonal regime. Extreme continental conditions prevailed, with hot summers and cold winters. The poles were ice-free, and polar sediments include coals, plants and spoils typical of cool temperate latitudes.

In the Late Permian, Pangaea was characterized by high average temperatures, and a very broad semiarid belt around the more humid Equator [50]. In many regions, aridity increased across the PTB and into the Early Triassic [16], with the northward and southward expansion of low-latitude arid belts into the vast formerly humid basins of European Russia and South Africa (figure 3). Primary sedimentological evidence of aeolian sediments, such as the preservation of ancient dunes, suggests an expansion through the PTB. Extensive areas of aeolian dune sandstones are reported from the Late Permian of the Paraná Basin of eastern South America, and then at the PTB from new locations, including the rift basins of Iberia, the south Urals and central Europe, in areas that had formerly been humid during the Late Permian [16]. This expansion of the arid belt was countered by increases in precipitation in other regions to balance the hydrological cycle.

Unusually, drought-killed associations of skeletons have been reported from the earliest Triassic of the Karoo Basin in South Africa [51]. The jumbled skeletons were interpreted as killed by drought around a shrinking water hole, and then aggregated by subsequent rainfall and transport. The drought interpretation is supported by the sedimentology of the sites, which shows lowering of water tables, the onset of drying through the loss of vegetation, the spread of vast playa lakes and accumulation of thin wind-blown laminae of loess dust.

Desiccation is an obvious stressor for plants and animals on land, frequently associated with high temperatures. All life depends on abundant water, so organisms show many adaptations to conserve water and minimize loss in adverse conditions [52]. Anti-desiccation adaptations are physiological, and behavioural for animals, which always seek to avoid extreme drying by moving to shade or foraging at night. Physiological adaptations include, in insects, for example, their impermeable cuticle with waxy components, tracheae with spiracles that can close and means to limit water excretion. Some insects pass into a dormant phase in desiccating conditions, where they choose a cool spot, reduce their body water content, decrease cuticular permeability, absorb water vapour and tolerate low body water levels. The chironomid midge *Polypedilum vanderplanki* is the largest multi-cellular animal known to survive almost complete dehydration without ill effect. Its larvae can tolerate an astonishing range of environmental temperatures, from −270 to +106°C, and it can recover after prolonged dehydration of up to 17 years. Part of the explanation seems to involve the rapid accumulation of the disaccharide sugar trehalose, which vitrifies, or forms an amorphous phase on desiccation, and so locks in water and other macromolecules.

**Figure 4.**
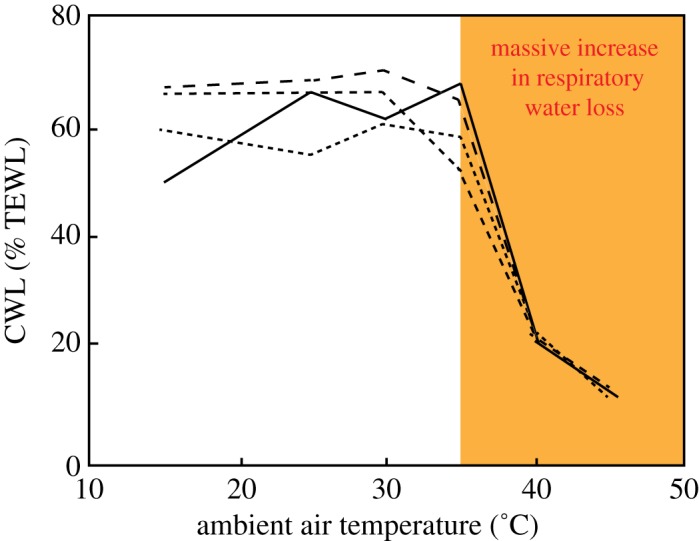
Cutaneous water loss (CWL) as a percentage of total evaporative water loss (TEWL) as a function of ambient air temperature for four species of larks—skylarks and woodlarks from The Netherlands, and hoopoe larks and Dunn's larks from Saudi Arabia. Based on data in [53]. (Online version in colour.)

Water loss through the skin is a crucial problem for animals. In insects, the biochemistry of their cuticle sets a critical limit beyond which water loss cannot be controlled. The same is true for vertebrates. During normal conditions, birds lose about 70% of water through their skin, but from 35 to 40°C, this breaks down and the rate of respiratory water loss increases exponentially (figure 4). In reptiles and birds, water loss through the skin is controlled by waterproofing lipids in the outer layer of the skin, the stratum corneum [53]. At normal skin temperatures, lipids form a dynamic mosaic in different phases, but primarily the orthorhombic phase, in which lipid molecules are packed close together and provide good waterproofing. As temperature rises above 40°C, lipids switch to the gel or liquid crystalline phase, and they are spaced further apart, providing holes in the lamellae through which water molecules can pass [53].

Simple organisms may be remarkably desiccation-tolerant, most notably certain bacteria and cyanobacteria, some of which can survive nearly complete removal of water, when the water content is less than 0.1 g H_2_O g^−1^ of dry mass. Such prokaryotes can sometimes survive complete drying for months or years, until water becomes available again. Desiccation tolerance is also shown by some metazoans, famously brine shrimp, tardigrades, rotifers and some insects. The brine shrimp *Artemia franciscana* produces embryonic cysts that can survive for 2 years in dry conditions, without oxygen, or even at temperatures below freezing or up to 80°C. In this species, the outermost envelope of the cyst is essential for its remarkable tolerance to stress. Tardigrades, or ‘water bears’, are arthropod-like microscopic animals that can survive in extreme environments, including temperatures close to absolute zero and as high as 151°C, massive doses of radiation and spans of up to 10 years without water [54]. Tardigrades have even survived the vacuum of outer space for a few days. Tardigrades resist desiccation by shrinking, infolding their cuticle, reducing transpiration and forming an inert cyst. They possess ‘intrinsically disordered proteins’ that form non-crystalline amorphous solids upon desiccation; in other words they vitrify, or form a glass-like substance, that locks in macromolecules and prevents damage under all kinds of physical environmental pressures [54]. Finally, rotifers, small (less than 2 mm) aquatic animals, are adapted to desiccation. Many live in temporary freshwater pools or terrestrial mosses or lichens, and when these habitats dry up, they can alter their morphology and physiology, assuming a compact dormant phase called a ‘tun’. Rotifers can survive annual cycles of drought, and the longest reported survival is 9 years. They then recover in minutes or hours when exposed to water. Rotifers, tardigrades and most other desiccation-resistant animals and plants possess the disaccharide sugar trehalose [55], as in certain insects.

As noted earlier, high temperatures and drought often go together, and adaptation to both stressors is commonplace in desert plants. Seeds are famous plant adaptations for surviving droughts, and many can remain viable for 5–10 years, and there are some credible records of 200-year-old seeds germinating [56]. Desiccation tolerance in seeds is promoted by mechanisms that prevent lethal damage to cellular components, including membranes, proteins and cytoplasm. There are three main protective systems [56]: the accumulation of non-reducing sugars such as trehalose, which stabilize membranes and proteins in dry conditions and lock in water in a glass phase in the cytoplasm; the ability to prevent, tolerate or repair a free-radical attack during desiccation; and the occurrence of protective proteins in late embryogenesis.

Among plants, bryophytes (mosses and liverworts) include many extreme survivors of desiccation; as an example, a dried herbarium specimen was reported to have regrown after 23 years of storage in entirely dry conditions [57]. Bryophytes differ from vascular plants in being smaller, having leafy shoots that equilibrate rapidly with the water potential in their surroundings and having shoots that tend to be either fully hydrated or desiccated and metabolically inactive. Bryophytes can suspend normal physiological processes when dried, and then respiration, photosynthesis and protein synthesis can recover in minutes or hours; recovery of the cell cycle, food transport and the cytoskeleton may take a day or more [57].

Vascular plants, including ferns, conifers and flowering plants, do not show such extremes of adaptation to desiccation. Indeed, the 300 species of angiosperms that can recover after severe desiccation are so unusual that they are termed ‘resurrection plants’ [58]; these belong to some 10 angiosperm families, and evolved their special properties convergently. Adaptations to desiccation [58] include modifications to the cell membranes and macromolecules, especially the production of large amounts of desiccation-induced protective proteins. These have different roles in cellular protection, by conserving the structures of macromolecules and membranes, by stabilizing membrane structures and proteins, by avoiding mechanical damage from vacuole shrinkage in dehydrating cells and by minimizing oxidative stress from the enhanced production of reactive oxygen. Resurrection plants can survive for months or years without water, and then regrow at full vigour when watered, but they do not survive for years.

From the fact that high temperatures and drought occurred through the time of the PTME, and knowledge about desiccation tolerance in modern organisms, it should be possible to explore how far drought contributed to the PTME killings. Studies of the physiology of modern microbes, plants and animals have shown that some clades are extremely desiccation-tolerant, and evidence could be sought whether the extinctions were selective. This would apply especially to some invertebrate and plant groups, if the stress lasted no more than 1–10 years, and where seeds or resting cysts could preserve the species long enough. Such desiccation tolerance might be restricted to small plants and animals, for example, plants less than 3 m and animals less than 5 mm, and particularly those animals with rigid skeletons [59]. Many desiccation-tolerant organisms share biochemical and cellular mechanisms, such as sugars that replace water and form glasses, proteins that stabilize macromolecules and membranes, and anti-oxidants that counter damage by reactive oxygen species [59]. These systems are often triggered by drying, and some of the genes involved may be homologous in microbes, plants and animals [56]. In that the drought was likely not worldwide during the PTME, this can have been only a regional killer, contributing to driving many plants and animals into smaller geographical areas that were free of excessive warmth and drought.

### (e) Acid rain as a killer on land

There is evidence that acid rain was a key consequence of the release of sulfur, chlorine, fluorine and other gases by the Siberian Traps eruptions [60]. This evidence comprises massive erosion of upland areas following the stripping of vegetation [16,26], mass wasting and supply of bursts of nutrient-rich soil and siliciclastic debris to circumcontinental shallow seas [27], expansion of the OMZ and boosts in shallow marine productivity [4,8,9,16]. These acid rain crises were probably repeated multiple times through the PTME crisis and subsequent Early Triassic events. The famous ‘coal gap’, lasting for the first 10 Myr of the Triassic, indicating the absence of trees and forests, is surely a measure of the impact of acid rain on terrestrial ecosystems. Acid rain generated by the Siberian Traps eruptions likely reached pH = 4 globally, and pH = 2 or 3 during the eruptions, and the rate of change was rapid [60], corresponding to likely drastic effects on life on land.

Acid rain damages plants in two ways, by direct contact and through leaching essential ions from the soil. Acid rain reduces the chlorophyll content of leaves and hence the ability of plants to photosynthesize. Other direct effects include physiological impacts (reduction in photosynthetic rate, variation in stomatal conductance and decrease in chlorophyll content) and morphological damage (decrease in thickness of cuticle, reduction in leaf area, discoloration and occurrence of necrotic spots). Further, the excess nitrogen and sulfur slows growth and increases susceptibility to stressors such as drought, frost, pest damage, disease and ozone increases [61].

Soil leaching may be more significant. Today, anthropogenic sulfur dioxide, together with ozone and nitrogen oxides, the key constituents of acid rain, have a direct effect on soil chemistry and forest health. This acid cocktail leaches base cations (Ca, Mg, K), increases the availability of soil Al and drives the accumulation and transmission of acidity from forest soils to streams. These changes in soil chemistry produce direct impacts on plants. Ca is an essential plant nutrient, contributing to many cellular structures and physiological processes as well as overall forest function. Ca uptake by plants can be inhibited by Al in solution, and these Ca-dependent metabolic and physiological processes are then disrupted. The ratio of Ca to Al in soil solution is used as a key indicator of forest health, especially in acid soils, and Ca loss points to acidification. Calcium reduction has profound effects on trees. Labile Ca, moving among cellular compartments, acts as a signal mediating physiological responses to environmental stresses such as drought, cold, heat, salinity, fungal pathogens, and oxidative and mechanical stresses [62]. Ca deficiency caused by acid rain can affect the ability of plants to sense and respond adaptively to their environment in several ways: it diminishes photosystem function by reducing leaf area; it reduces carbohydrate metabolism by reducing the storage of sugars; it reduces the cold tolerance of leaves, and so increases the risk of winter injury and crown deterioration; it impairs the function of the leaf stomata, essential for correct water balance; and it affects seed germination and seedling growth.

Whereas today much of total global biodiversity resides on land and in angiosperm forests, the balance of biodiversity was probably different in the Permian and Triassic, with less life on land [63]. Nonetheless, trees then must have harboured considerable biodiversity, and so the stripping of vegetation from the land, including forests, would have removed specialized habitats for many arthropods and vertebrates. The arrival of acid rain during the PTME was fast and likely repeated during each major eruption event, and the stripping of forests and mass wasting by the most extreme acid rain events would have had devastating effects on all life on land.

### (f) Wildfires as killers on land

There is considerable evidence for prevalent wildfires at the time of the PTME. These would have looked spectacular, like the lava flows around the Siberian Traps eruptions, but their role in causing global extinction might have been modest. Forest fires happen all the time, and may devastate ecosystems locally or regionally, but life soon recovers and heals the scars.

In fact, wildfires were common throughout the Permian and right through to the PTB, and then there is a long gap during the Early Triassic and much of the Middle Triassic, when fossil charcoal is not reported, or was at least very rare, as were other indicators of fire, including inertinites and pyrogenic polycyclic aromatic hydrocarbons [64]. Wildfire has been posited at the PTB at Meishan in South China, based on abundant charcoal, black carbon and carbon spherules [14]. Wildfire can kill directly as forests were burnt down, but most animals would have been able to flee. Wildfires contribute to extinction also by releasing trace gases and particulates into the atmosphere that can in turn influence atmospheric chemistry and climate. Further, wildfires remove forests and so enable mass wasting and wash-off of sediments down rivers and into lakes and marginal seas.

Today, many ecosystems are beset by frequent wildfires, and yet they recover. Australia today is perhaps the most fire-affected continent, and large proportions of the northern tropical savannah landscapes are burnt [65]. Yet, much of the savannah biota is remarkably resilient to fire, even high-intensity fire. Fires make little difference to the relative abundances of plants and animals, except for riverbank plants and animals, and small mammals. The 2-yearly cycle of managed burning appears to be too frequent for small mammals, which have undergone significant declines. Overall, however, life recovers from such major fires, and fire seems to be a natural process in such areas.

Counterintuitively perhaps, fire can increase biodiversity. Fires can act as a disturbance, preventing species of late successional stages from excluding those of earlier stages. Fires can therefore increase biodiversity by preventing any single species from dominating. In experimental field studies, observing either natural or artificial fires, the frequency of fires can increase the richness of species that live deep within forests and at forest edges, but species of open landscapes, open forests and interior forests were not influenced by fire frequency. Fire also had a positive impact on animal diversity for most subclades of insects and spiders, but reduced biodiversity of isopods and weevils, some of which prefer damp habitats in dead wood. Flying arthropods proved to be most resilient to fire, then pollen-feeders and foragers, and ground-litter scavengers were least able to recover following major fires.

There are three reasons that wildfires, although they may have been prevalent at the time of the PTME, were probably minor killers: (i) wildfires had been common throughout the Permian, and there is limited evidence they were larger or more devastating at the PTB than earlier; (ii) effects of wildfires can be quickly repaired; and (iii) fire can in fact increase biodiversity, rather than simply reduce it.

### (g) Ozone destruction as a killer on land

The ozone shield around the atmosphere may have been destroyed at the end of the Permian by one of three atmospheric gases: halogens, hydrogen sulfide or organohalogens. The initial suggestion was injection of chlorine and fluorine into the atmosphere from the Siberian Traps eruptions [60,66]. Alternatively, or in addition, hydrogen sulfide and methane generated in the euxinic oceans might have leaked into the atmosphere and had the same effect, although that has been questioned [66,67]. Another possible cause of breakdown of the ozone layer was the postulated release of large quantities of organohalogens from the heating of organic-rich rocks and hydrothermal fluids, particularly by the passage of molten lavas of the Siberian Traps through thick coal deposits [66]. The ozone layer would have been penetrated at high latitudes, but not around the Equator because of its ‘self-healing’ properties. Modelling results [60] suggest that methane and methyl chloride fluxes triggered by the Siberian Traps eruptions could have reduced ozone production by 60–70% globally, leading to short-term increases in UV light flux of 400% around the Equator and up to 5000% at the poles.

The key evidence for destruction of the ozone layer during the PTME is the appearance of mutated spores and pollen in some rock sections. These were interpreted as having been affected by increased UV-B radiation reaching the Earth from the Sun through an ozone hole, and disrupting normal meiotic (cell division) processes. However, if such mutations are not solely the product of UV-B radiation, and modern experiments show that such effects can be induced by high levels of sulfur dioxide [67], then it is less certain that the ozone layer did break down during the PTME.

An analysis [68] of malformed spores and pollen grains, unseparated tetrads and darkened walls of spores and pollen (sporoderm) from Permian–Triassic sediments of the Finnmark Platform of offshore Norway concluded that the damage might have been caused by a breakdown in the ozone layer, but more likely by increased heavy-metal ions, arsenic, organohalogens and acid rain (SO_2_). Perhaps the ozone layer did not break down, or perhaps it did not drive terrestrial extinctions in any major way.

## 5. Conclusion

Hyperthermal events, such as at the PTME and the PETM, and many others through the Permian to Jurassic interval, when massive volcanic eruptions triggered sharp global warming, produce a series of perhaps predictable effects. For organisms in the sea, killers were the combined effects of extreme warming acting from above and euxinia from below to produce a narrow to absent habitable depth zone [31]. On land, the killing cocktail was rather different, with an emphasis on acid rain destroying habitats, associated with drought and extreme warming, which halved the habitable area. Wildfires and breakdown of the ozone layer might have contributed to the terrestrial extinctions, or might have had negligible effects. In both cases, hypoxia may have been less of a stressor than had been suggested because of the likely slow rates of change of atmospheric and oceanic oxygen levels.

Hyperthermals have been identified as killers during many Phanerozoic events, especially from the Permian, Mesozoic and Palaeogene. It is important, however, to consider each event in the context of longer-term conditions. The fact that the Permian–Triassic was already a hothouse world may have been significant. In fact, the PTME events exacerbated certain long-term processes, as noted, including increasing temperatures, increasing aridification and reducing atmospheric oxygen throughout the Permian, and continuing into the Triassic [16]. When hyperthermals strike during ice-house conditions, the effects and impacts on life might be different.

Further, some of the consequences of major eruption at the PTB, including global warming, acid rain, mass wasting and marine anoxia, may have been repeated two or three times during the Early Triassic when comparable isotopic perturbations occurred [67]. Certainly, there is evidence for repeated episodes of mass wasting and input of siliciclastic debris into the sea during the Early Triassic, so suggesting repeated episodes of acid rain [27].

If acid rain, mass wasting and aridity were killers of life on land during the PTME, it ought to be possible to distinguish differences in survival rates between those species that were most likely to have been drought-resistant compared to those that were not. In broad terms, insects and bryophytes might then be expected to have shown higher survival than vertebrates and vascular plants. The difficulty in carrying out a satisfactory meta-analysis of this kind is that many desiccation-tolerant clades, such as rotifers, tardigrades and bryophytes, have poor fossil records, and there is no evidence for measurable differences in drought resistance between major clades of plants, insects and vertebrates that were either victims or survivors of the PTME.

## Data accessibility

This article has no additional data.
